# The application of Chinese version of SARC-F and SARC-CalF in sarcopenia screening against five definitions: a diagnostic test accuracy study

**DOI:** 10.1186/s12877-024-05460-w

**Published:** 2024-10-26

**Authors:** Jia-Yu Guo, Kang Yu, Chun-Wei Li, Yuan-Yuan Bao, Yu Zhang, Fang Wang, Rong-Rong Li, Hai-Yan Xie

**Affiliations:** 1 Department of Clinical Nutrition, Peking Union Medical College Hospital, Chinese Academy of Medical Sciences (CAMS) and Peking Union Medical College (PUMC), Beijing, 100730 China; 2 Department of Health Care, Peking Union Medical College Hospital, Chinese Academy of Medical Sciences (CAMS) and Peking Union Medical College (PUMC), Beijing, China

**Keywords:** Sarcopenia, SARC-F, SARC-CalF, Validation, Diagnostic test

## Abstract

**Background:**

SARC-F questionnaire is a simple and convenient tool for sarcopenia screening, and SARC-CalF is a modified version of it. The developments of their Chinese versions are warranted for the clinical use for Chinese population. This study aimed to culturally adapt the SARC-F questionnaire into Chinese using standardized methods, validate the reliability and diagnostic accuracy of the Chinese version SARC-F and SARC-CalF against five sarcopenia diagnosis criteria, and determine optimal cut-off values for clinical practice in Chinese population.

**Methods:**

The translation and cross-cultural adaptation of SARC-F into Chinese were conducted following the methodological report from European Union Geriatric Medicine Society Sarcopenia Special Interest Group. The Chinese version of SARC-F was validated through a diagnostic test, using diagnostic criteria of sarcopenia recommended by the revised 2019 European Working Group on Sarcopenia in Older People (EWGSOP2) consensus, Asian Working Group for Sarcopenia (AWGS2019) consensus, the International Working Group on Sarcopenia (IWGS), the Foundation for the National Institutes of Health (FNIH) Biomarkers Consortium and the Sarcopenia Definition and Outcomes Consortium (SDOC). Additional analysis was done against the criteria of severe sarcopenia according to the revised EWGSOP2 and AWGS2019.

**Results:**

The Chinese version of SARC-F was well translated and demonstrated good reliability and acceptability. The diagnostic test included 1859 community-dwelling older individuals from two medical centers. Against five different definitions of sarcopenia, the Chinese version of SARC-F showed reasonable diagnostic accuracy for sarcopenia screening (AUC 0.614–0.821), and was demonstrated low sensitivity (13.7–37.9%) but high specificity (94.8–97.7%) with a cut-off value of ≥ 4. SARC-CalF significantly enhanced the diagnostic accuracy of SARC-F when using definitions of EWGSOP2, AWGS2019 and IWGS (all *P* ≤ 0.001). A score of ≥ 2 for SARC-F and ≥ 7 for SARC-CalF were established as optimal cut-off points for identifying older individuals as at risk of sarcopenia in Chinese population.

**Conclusions:**

The Chinese version SARC-F is of reasonable reliability and validity for sarcopenia screening. Despite its low sensitivity, it proves to be a useful tool to identify severe cases in community taking advantage of its simplicity. SARC-CalF appears to be a more suitable screening tool for clinical use in detecting sarcopenia.

**Supplementary Information:**

The online version contains supplementary material available at 10.1186/s12877-024-05460-w.

## Background

Sarcopenia was initially proposed by Irwin H. Rosenberg as the age-related loss of skeletal muscle mass [1]. Currently, sarcopenia has emerged as a nonnegligible geriatric syndrome affecting 10–27% of individuals ≥ 60 years worldwide [2]. It has been recognized as a disease entity with the awarding of an ICD-10‐CM (M62.84) code in 2016 [3]. Substantial efforts have been invested in elucidating the definition and diagnosis criteria of sarcopenia. The algorithm recommended by the revised 2019 European Working Group on Sarcopenia in Older People (EWGSOP2) consensus encompasses case-finding, diagnosis and severity quantification of sarcopenia, facilitating a straightforward and systematic process in the application of clinical setting [4]. The importance of sarcopenia screening is increasingly recognized, particularly through self-reporting by at-risk populations. The early sarcopenia detection can improve their compliance with lifestyle interventions achieved by integrating individuals into a multidisciplinary team system.

SARC-F questionnaire is such an economical and convenient tool for sarcopenia screening for both proxy-report and self-report with only five simple questions [5, 6]. This tool was recommended by EWGSOP2 and Asian Working Group for Sarcopenia (AWGS2019) for case-finding in clinical practice [4, 7]. The SARC-F questionnaire has been translated into multiple languages and validated across diverse populations following the methodological suggestions outlined by the European Union Geriatric Medicine Society Sarcopenia Special Interest Group [8]. The modified version of SARC-F, called SARC-CalF, was developed by incorporating the calf circumference (CC) measurement to enhance the sensitivity and the area under the receiver operating characteristics (ROC) curve (AUC) without compromising the remaining parameters [9].

Due to the accelerating aging in Chinese society, sarcopenia has emerged as a growing problem with a prevalence of 9.2–16.2% in China based on our prior epidemiological study [10]. It is of great importance to focus on the early detection of sarcopenia in Chinese older populations. Therefore, SARC-F needs to be adapted and validated in Chinese cultural context, to support future studies, and to promote early detection and intervention for sarcopenia among Chinese population. At present, the questionnaire used in Chinese population lacks specific validation, and moreover the simple translation does not ensure cultural and conceptual equivalence. This study aimed to culturally adapt the original SARC-F questionnaire into Chinese using standardized methods, to validate the reliability and diagnostic accuracy of the Chinese version SARC-F and SARC-CalF against various sarcopenia diagnosis criteria, and to determine the optimal cut-off values clinical practice in Chinese population.

## Methods

### Study design and population

We used the cross-sectional data between December 2021 and October 2022 from the Peking Union Medical College Hospital Multicenter Prospective Longitudinal Sarcopenia Study, which is an ongoing nationwide interdisciplinary cohort study to evaluate changes in muscle and clinical outcomes among older people in China [11] and was registered at clinicaltrials.gov as NCT02873676. The community-dwelling participants were recruited from communities and hospitals using simple random sampling from two medical centers in Beijing and Tianjin. The study protocol was approved by the Human Ethics Committee of the PUMC Hospital (no. ZS-3462). All patients participating in this study provided written informed consent.

Participants aged 60 to 85 years, with physical and/or mental capacity to perform the requested tests, were included in the study. Participants with infectious diseases, neuromuscular diseases (e.g., myasthenia gravis), implants of electronic devices or metal materials, and edema or anatomical deformities in lower extremities were excluded from the study.

### Measurements

Face-to-face interviews, anthropometry and body composition measurements were conducted by trained investigators. And a structured paper-and-pencil questionnaire was completed by the investigators to obtain information, including demographic characteristics, dietary intake and nutritional status, physical activity, comorbidities, and frailty status. Demographic characteristics contained age, gender, region, smoking status and drinking status. The anthropometry and body composition measurements included height(m), weight(kg), grip strength(kg), waist circumference(cm), CC(cm), appendicular muscle mass(kg), gait speed(m/s).

Trained investigators asked each participant to report the frequency and the usual amount of consumption of each food item over the past year, and the amount of dietary protein intake(g/d) was estimated by dietitians based on the Database of Chinese Food Composition. Nutritional status was assessed using the Mini Nutritional Assessment-Short Form (MNA-SF) with a total score of 0–14, classifying patients into normal nutritional status, malnutrition risk and malnourished groups if scores of 12–14, 8–11 and 0–7 points, respectively [12]. The International Physical Activity Questionnaire-Short Form (IPAQ-SF) was used to categorize the participants as high, moderate and low intensity of physical activity by recording walking, moderate-intensity and vigorous-intensity activity in the past 7 days [13]. Comorbidities, including hypertension, coronary heart disease, diabetes, respiratory disease and cancer were assessed by referring to the self-reported physician’s diagnosis. Frailty status was defined using Fried’s frailty phenotype criteria, dividing the participants into non-frailty, pre-frailty and frailty when 0, 1–2 and ≥ 3 of the following criteria were present: unintentional weight loss, self-reported exhaustion, weakness, slow walking speed, and low physical activity [14].

Waist circumference was measured midway between the lateral lower rib margin and the superior anterior iliac crest at the end of a gentle expiration phase. CC was measured on the left leg in a seated position with the knee and ankle at right angles, feet resting on the floor using a nonelastic tape. The mean of 2 measurements of CC was considered. Grip strength was measured by using a digital hand dynamometer (CAMRY MODEL EH101, HaNDCReW, Guangdong, China). Two consecutive measures of grip strength in the dominant hand were recorded, with the participant in a standing position and the arm of measured hand placed parallel to the body, as recommended by AWGS2019 consensus [7]. Grip strength was calculated by taking the average of the two measurements. Gait speed was measured by timing the participants’ ability to walk 6 m at a normal pace. Each participant performed the gait speed assessment twice, with the faster of the two times used as the representative score, as recommended by AWGS2019 consensus. Low gait speed was defined as gait speed < 1.0 m/s [7].

Muscle mass was measured using a segmental multifrequency bioelectrical impedance analysis (M-BIA) instrument (H-Key350, Beijing Seehigher Technology Co., Ltd). Participants were instructed to fast for 12 h and avoid vigorous exercise before the measurements, as well as dressed in light clothes and were barefoot. The appendicular skeletal muscle mass index (ASMI) was calculated by dividing the sum of total appendicular skeletal muscle mass (ASM) in kilograms by height in meters squared. ALM_BMI_ was calculated by dividing the sum of total appendicular lean mass (ALM) in kilograms by body mass index (BMI).

### Diagnosis of sarcopenia

Sarcopenia was defined based on the diagnostic criteria endorsed by the consensus of EWGSOP2 [4], AWGS2019 [7], the International Working Group on Sarcopenia (IWGS) [15], the Foundation for the National Institutes of Health (FNIH) Biomarkers Consortium [16] and the Sarcopenia Definition and Outcomes Consortium (SDOC) [17]. Severe sarcopenia was determined according to the criteria of EWGSOP2 and AWGS2019 [4, 7].

According to the EWGSOP2 definition, sarcopenia was characterized by low muscle strength (grip strength M:<27 kg, F:<16 kg) and reduced muscle mass (ASMI M:<7.0 kg/m^2^, F:<5.5 kg/m^2^). Severe sarcopenia was defined as reduced muscle mass, low muscle strength and low physical performance (gait speed ≤ 0.8 m/s) [4].

According to the AWGS2019 definition, sarcopenia was defined as reduced muscle mass (ASMI M:<7.0 kg/m^2^, F:<5.7 kg/m^2^) and either low muscle strength (grip strength M:<28 kg, F:<18 kg) or low physical performance (gait speed < 1.0 m/s). Severe sarcopenia was identified in participants with reduced muscle mass, low muscle strength and low physical performance [7].

According to the IWGS definition, sarcopenia was characterized by reduced muscle mass (ASMI M: ≤7.23 kg/m^2^, F: ≤5.67 kg/m^2^) and low physical performance (gait speed < 1.0 m/s) [15].

According to the FNIH definition, sarcopenia was defined as reduced muscle mass (ALM_BMI_ M:<0.789, F:<0.512), low muscle strength (grip strength M:<26 kg, F:<16 kg) and low physical performance (gait speed ≤ 0.8 m/s) [16].

According to the SDOC definition, sarcopenia was identified in participants with both weakness (grip strength over BMI M: <1.05, F: <0.79) and slowness (gait speed < 0.8 m/s) [17].

### Screening of sarcopenia risk

The SARC-F questionnaire was designed as a quick tool for sarcopenia screening consisting of five components: strength, assistance with walking, rising from a chair, climbing stairs and falls [5]. It has an original cut-off value of ≥ 4 for at risk of sarcopenia. We utilized the Chinese version of SARC-F questionnaire, which was translated and cross-cultural adapted through the standardized process, as detailed in Table S1 and S2. The SARC-CalF questionnaire combined the five components of SARC-F and CC by directly summing their scores. The CC component in SARC-CalF additionally scores 10 points, with cut-off values of ≤ 34 cm for men and ≤ 33 cm for women, otherwise scores 0 points [9]. It has an original cut-off value of ≥ 11 for at risk of sarcopenia. In our study, the participants answered the questions of SARC-F by themselves with the investigators recording the scores.

### Translation, cross-cultural adaptation and validation of Chinese version SARC-F

The translation and cross-cultural adaptation of SARC-F in Chinese were performed according to the standardized methods recommended by World Health Organization and the methodological report from European Union Geriatric Medicine Society Sarcopenia Special Interest Group [8, 18].

The translation and cross-cultural adaptation process involved forward translation, expert panel, back-translation, pretest and cognitive interviewing and the determination of the final version. In the forward translation step, a bilingual translator translated the original SARC-F questionnaire into Chinese. Subsequently, the expert panel, consisting of two nutrition and one geriatrician bilingual experts and the forward translator, reviewed the translation and expressions, engaging in discussions to formulate the reconciled version. The subsequent back translate process was conducted by another bilingual translator proficient in English and blinded to the original questionnaire. A pretest included 10 participants from target population was conducted through personal interviews. The demographic characteristics of age, gender and education level were collected. Participants were administered the translated instrument and asked to confirm their understanding of each question and to repeat it in their own words. The consistency of the answers to the instrument and questions was assessed. According to the back translate version and pretest results, the expert panel discussed with the translators to formulate the final version of Chinese version SARC-F questionnaire.

Both a pilot study for reliability validation and a cross-sectional study for clinical validation were conducted to validate the Chinese version SARC-F. In the pilot study, assessments were made for the test-retest reliability, inter-rater reliability and internal consistent reliability. Twenty participants from the target population were admitted to the pilot study. They were required to self-complete the Chinese version SARC-F and were then reassessed by another investigator blinded to the self-reported answers. After two weeks, another repeated test took place. The time of each test was recorded simultaneously. Through the following cross-sectional study, the diagnostic test accuracy of the Chinese version SARC-F would be assessed, against the five definitions of sarcopenia (EWGSOP2, AWGS2019, IWGS, FNIH and SDOC), as well as against severe sarcopenia of EWGSOP2 and AWGS2019.

### Statistical analysis

The database was established using EpiData 3.1 software and the statistical analysis was performed by STATA/SE 16.0 Software. Continuous variables were described as means ± SD or medians (interquartile ranges), and the categorical variables were described as counts and percentages. The comparisons between groups were analyzed using t-test, chi-squared test, Fisher exact test and Mann-Whitney U test, where appropriate. Spearman’s correlation coefficients between the score of SARC-F or SARC-CalF, and muscle-related parameters were calculated. Diagnostic tests were performed to validate the accuracy of Chinese version SARC-F and SARC-CalF, and were displayed by sensitivity, specialty, positive predictive value (PPV) and negative predictive value (NPV). The ROC analyses were conducted to calculate the AUC. Youden index method was used to determine the optimal cut-off point of SARC-F or SARC-CalF, and calculated by sensitivity and specificity. Differences were considered significant at *P* < 0.05.

For sample size calculations, we took the sensitivity of 27% and specificity of 91% from previous studies [19], with a power of 95% and a target significance level of 5%. Taking 11.6% as the prevalence of sarcopenia in this population [10], the sample size of the clinical validation was calculated as 1845 subjects.

## Results

### Translation and cultural adaption of Chinese version SARC-F

In the pretest phase, we included 10 participants aged 61 to 85 years, with equal representation of men and women and 60% having education level below senior high school. During the interview, all the participants demonstrated an understanding of the meaning of each question and could rephrase the questions in their own words. Their responses to the instrument and the interview questions were consistent.

As shown in Table S1 and S2, The Chinese version SARC-F questionnaire and the back translated version were finally determined, and were approved by John Morley, one of the authors of the SARC-F questionnaire, confirming that the translation could convey the original meaning accurately. For cultural adaptation, the weight of 10 pounds in the first question was converted to SI unit (4.5 kg), with an annotation of 9 jin (a unit of weight used in China, 1 jin = 0.5 kg).

### Reliability validation of Chinese version SARC-F

The reliability and acceptability of the Chinese version SARC-F were analyzed in the pilot study with 20 participants meeting the inclusion criteria. Results showed that the questionnaire recovery rate was 100%, and the completion rate was 100%. The test-retest reliability between two weeks had a high intraclass correlation coefficient (ICC) of 0.91 (95% confidence interval [CI] 0.75–1.07). The answers of self-assessment and proxy-assessment by the trained investigators had a strong correlation (*r* = 0.73, *P* = 0.0003), indicating the high inter-rater reliability. The internal consistent reliability had the Cronbach’s alpha of 0.73, indicating a high level of internal consistency.

The time required to complete the Chinese version SARC-F ranged from 0.5 to 6.5 min, with an average of 2.11 ± 1.26 min. The average time taken for self-assessment was 2.11 ± 1.22 min, and for assessment by the trained investigators was 2.12 ± 1.32. The difference between the two groups had *t* = 0.02 (*P* = 0.9804), indicating no statistical difference between self- assessment and proxy-assessment times.

### Clinical validation of Chinese version SARC-F and SARC-CalF

#### Population and characteristics

After the exclusion of 65 participants (*n* = 40 for missing data of body composition or anthropometry measurement, *n* = 24 for missing data of SARC-F assessment, *n* = 1 for duplicate entries), 1859 participants were included in the statistical analysis. In the clinical validation study, the recovery rate of the Chinese version SARC-F was 86.3%, and the completion rate was 99.0%, with the Cronbach’s alpha of 0.7405. Table 1 displays the basic characteristics of the included participants. Among the 1859 participants, 838 males counted for 45.08%, and 91.34% of participants live in rural areas. These participants had an average age of 71.18 ± 4.92 years.

**Table 1:** Characteristics of the study population

Characteristics		Total (*N* = 1859)	Male (*N* = 838)	Female (*N* = 1021)
Age (y) (mean ± SD)		71.18 ± 4.92	71.17 ± 5.02	71.19 ± 4.84
Region (n [%])	Rural	1698 (91.34)	739 (88.19)	959 (93.93)
	Urban	161 (8.66)	99 (11.81)	62 (6.07)
Smoking (n [%])	No	1443 (77.62)	469 (55.97)	974 (95.40)
	Yes	416 (22.38)	369 (44.03)	47 (4.60)
Drinking (n [%])	No	1383 (74.39)	429 (51.19)	954 (93.44)
	Yes	476 (25.61)	409 (48.81)	67 (6.56)
Comorbidity (n [%])	None	554 (29.80)	291 (34.73)	263 (25.76)
	Hypertension	945 (50.83)	388 (46.30)	557 (54.55)
	CHD	174 (9.36)	62 (7.40)	112 (10.97)
	Diabetes	353 (18.99)	138 (16.47)	215 (21.06)
	Respiratory disease	22 (2.63)	10 (0.98)	32 (1.72)
	Cancer	55 (5.39)	136 (7.32)	81 (9.67)
IPAQ (n [%])	Low intensity	88 (10.5)	198 (19.39)	286 (15.38)
	Moderate intensity	571 (68.14)	698 (68.36)	1269 (68.26)
	High intensity	179 (21.36)	125 (12.24)	304 (16.35)
MNA-SF stage (n [%])	Malnourished	12 (1.43)	5 (0.49)	17 (0.91)
	Malnutrition risk	159 (18.97)	210 (20.57)	369 (19.85)
	Normal	667 (79.59)	806 (78.94)	1473 (79.24)
Fried frailty phenotype (n [%])	Non-frailty	245 (29.24)	251 (24.58)	496 (26.68)
	Pre-frailty	502 (59.9)	586 (57.39)	1088 (58.53)
	Frailty	91 (10.86)	184 (18.02)	275 (14.79)
Low gait speed (n [%])		368 (43.91)	605 (59.26)	973 (52.34)
Grip (kg) (mean ± SD)		20.72 ± 7.66	25.90 ± 7.08	16.47 ± 5.05
ASMI (kg/m^2^) (mean ± SD)		6.94 ± 0.99	7.60 ± 0.81	6.39 ± 0.78
BMI (kg/m^2^) (mean ± SD)		25.03 ± 3.59	24.60 ± 3.30	25.38 ± 3.78
SARC-F score (median [iqr])		0 (1)	0 (0)	0 (2)
SARC-CalF score (median [iqr])		1 (10)	0 (10)	2 (10)

Notes Continuous variables were described as means ± SD or medians (interquartile ranges[iqr]), and the categorical variables were described as count (percentage)

Abbreviations CHD, Coronary heart disease; IPAQ, The International Physical Activity Questionnaire; MNA-SF, The Mini Nutritional Assessment-Short Form; ASMI, Appendicular skeletal muscle mass index; BMI, Body mass index

#### Prevalence of sarcopenia according to different criteria

**Fig. 1:**
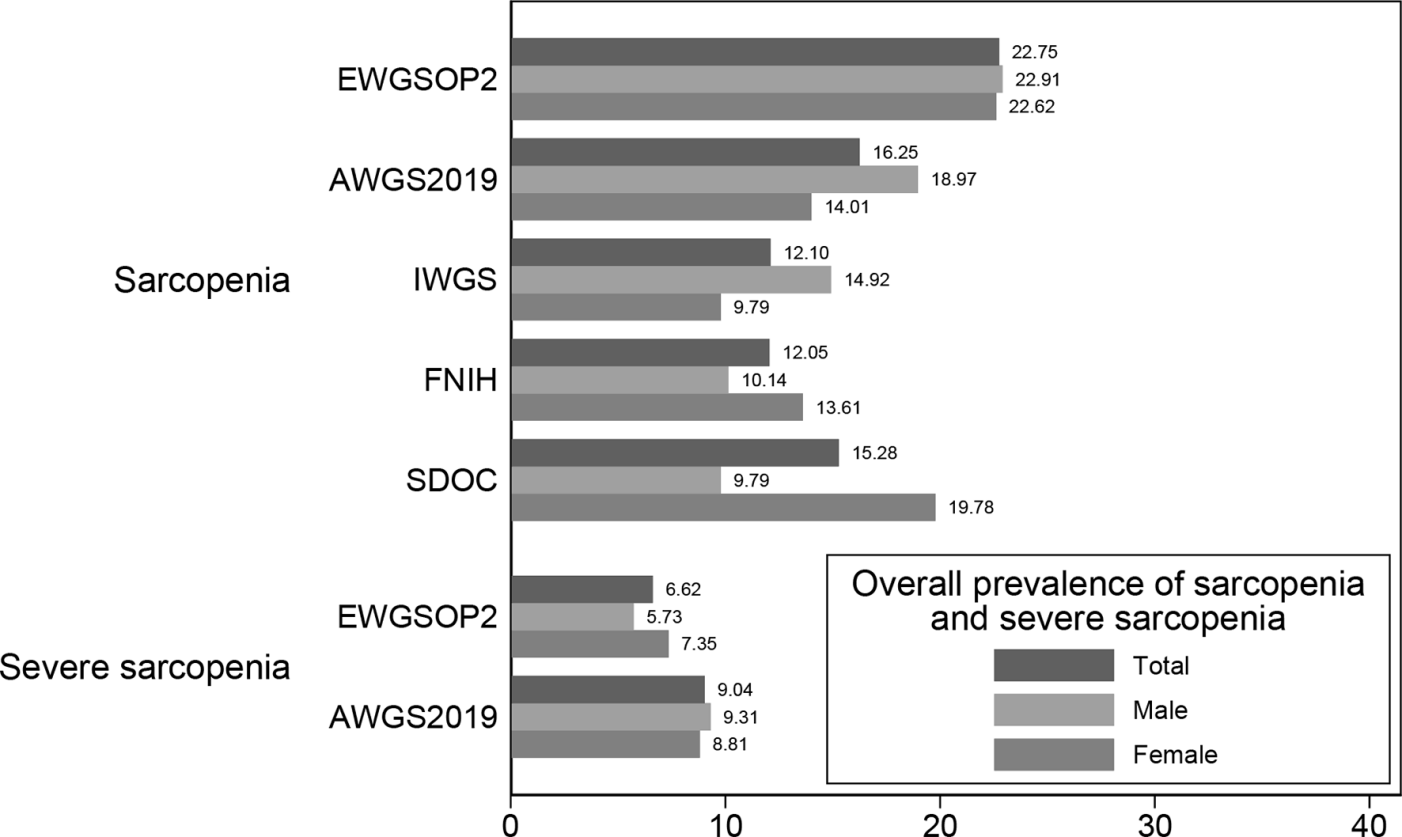
Overall prevalences of sarcopenia and severe sarcopenia according to different diagnostic criteria (%)

Figure 1 shows the overall prevalence of sarcopenia and severe sarcopenia in the study population. Based on the five diagnostic criteria, the overall prevalences of sarcopenia varied between 22.75% (EWGSOP2) to 12.05% (FNIH). The overall prevalences of severe sarcopenia across two criteria in both genders were less than 10%. For the Chinese version of SARC-F and SARC-CalF, the positive rates were 6.89% and 13.82% using cut-off values of 4 and 11, respectively.

#### Correlation between SARC-F or SARC-CalF and muscle-related parameters

**Fig. 2:**
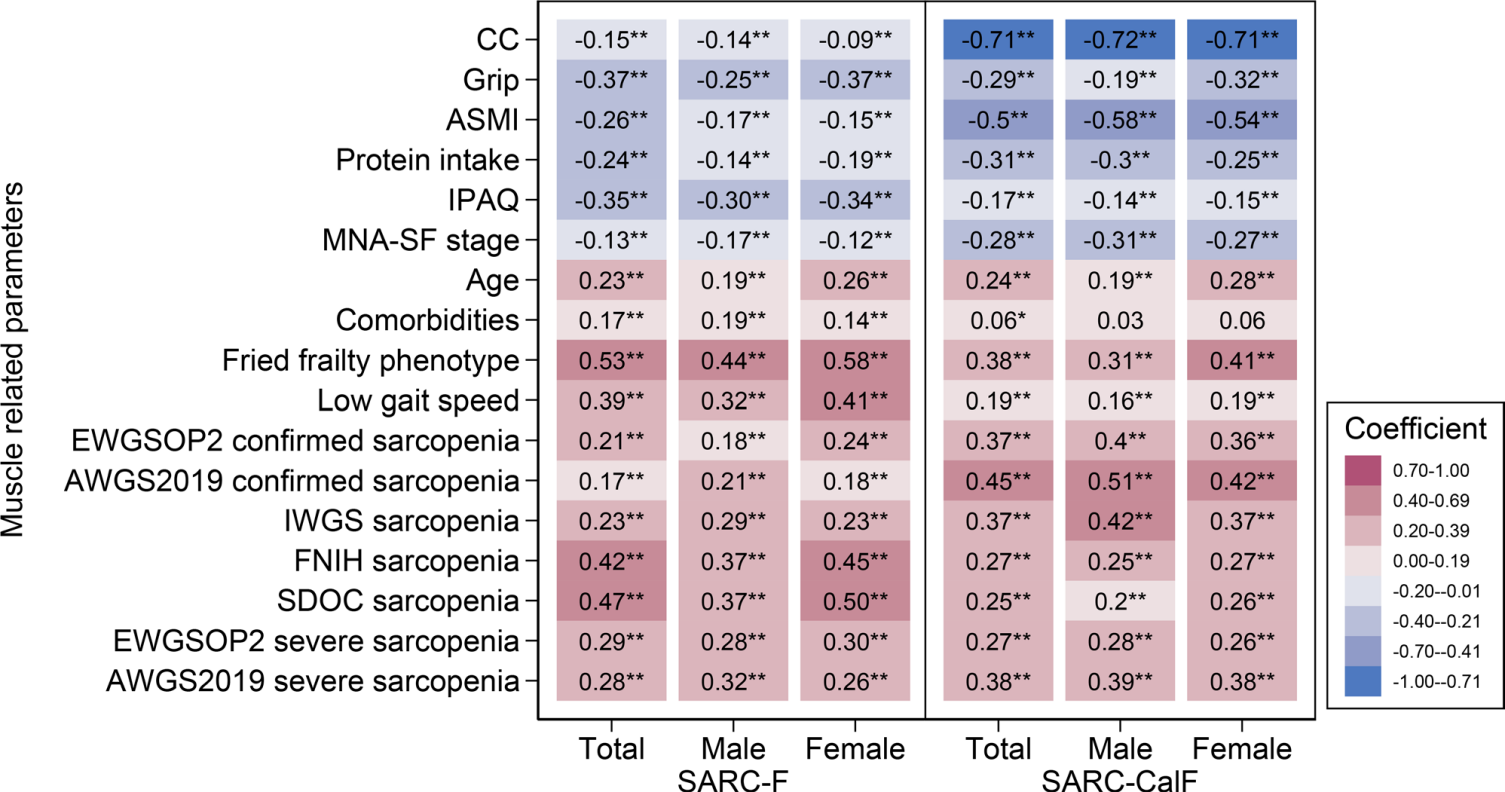
The Spearman’s correlation coefficient between SARC-F score or SARC-CalF score and muscle-related parameters. Notes: **Significantly correlated (*P* < 0.01). * Significantly correlated (*P* < 0.05). The coefficient *r*_*s*_ means a very weak correlation when 0 < *r*_*s*_≤0.2, a weak correlation when 0.2 < *r*_*s*_≤0.4, a moderate correlation when 0.4 < *r*_*s*_≤0.7, and a strong correlation when *r*_*s*_>0.7 (positive or negative)

Figure 2 shows Spearman’s correlation coefficient of SARC-F or SARC-CalF score and muscle-related parameters. Both scores positively correlated with higher age, more comorbidities, lower gait speed, frailty, sarcopenia and severe sarcopenia, while negatively correlating with higher CC, grip strength, ASMI, protein intake, more physical activities and better nutritional status. Chinese version SARC-F score is moderately positive correlated with frailty status (*r*_*s*_ [95%CI] = 0.53 [0.50–0.56], *P* < 0.01), FNIH defined sarcopenia (*r*_*s*_ [95%CI] = 0.42 [0.38–0.45], *P* < 0.01) and SDOC defined sarcopenia (*r*_*s*_ [95%CI] = 0.47 [0.43–0.51], *P* < 0.01). While Chinese version SARC-CalF score is moderately positive correlated with AWGS2019 defined sarcopenia (*r*_*s*_ [95%CI] = 0.45 [0.41–0.48], *P* < 0.01), and moderately negative correlated with muscle mass represented by ASMI (*r*_*s*_=-0.50, 95%CI=[-0.54]-[-0.47], *P* < 0.01).

#### Screening accuracy of the Chinese version SARC-F and SARC-CalF

Table 2 presents the diagnostic accuracy of SARC-F and SARC-CalF under the five diagnostic criteria of sarcopenia and under EWGSOP2 and AWGS2019 defined severe sarcopenia, shown as AUCs. These AUCs indicated no significant difference between genders, except a significantly higher AUC in female than male when diagnosing sarcopenia using FNIH definition (*P* = 0.036).

Among the five diagnostic criteria of sarcopenia, the SARC-F had the highest diagnostic accuracy (AUC [95%CI] = 0.821 [0.792–0.849]) when using SDOC definition as reference criteria, while the SARC-CalF had the highest diagnostic accuracy (AUC [95%CI] = 0.830 [0.806–0.854]) when using AWGS2019 definition as reference criteria. SARC-CalF consistently demonstrated significantly higher AUCs than SARC-F with all the *P* values for equality of ≤ 0.001, when screening for sarcopenia using the definitions of EWGSOP2, AWGS2019 and IWGS.

For severe sarcopenia, the SARC-F and SARC-CalF had comparable AUCs of 0.787 and 0.795 using EWGSOP2 definition (*P* = 0.709), while the SARC-CalF had a higher diagnostic accuracy (AUC [95%CI] = 0.858 [0.831–0.885]) when using AWGS2019 definition.

**Table 2:** The AUC of SARC-F and SARC-CalF against sarcopenia and severe sarcopenia

Diagnostic criteria	SARC-F				SARC-CalF				*P*_2_
	Total	Male	Female	*P*_1_	Total	Male	Female	*P*_1_	
**Sarcopenia**									
EWGSOP2	0.622 (0.593–0.650)	0.595 (0.556–0.634)	0.648 (0.609–0.688)	0.059	0.743 (0.716–0.769)	0.747 (0.707–0.787)	0.740 (0.705–0.775)	0.789	< 0.001
AWGS2019	0.614 (0.581–0.647)	0.618 (0.575–0.661)	0.633 (0.584–0.682)	0.648	0.830 (0.806–0.854)	0.842 (0.808–0.875)	0.836 (0.804–0.868)	0.802	< 0.001
IWGS	0.674 (0.637–0.711)	0.677 (0.628–0.726)	0.701 (0.646–0.755)	0.533	0.813 (0.783–0.843)	0.807 (0.765–0.85)	0.843 (0.809–0.878)	0.203	< 0.001
FNIH	0.814 (0.781–0.847)	0.765 (0.706–0.824)	0.839 (0.802–0.877)	0.036	0.724 (0.692–0.756)	0.722 (0.665–0.779)	0.716 (0.677–0.755)	0.880	< 0.001
SDOC	0.821 (0.792–0.849)	0.772 (0.714–0.831)	0.828 (0.796–0.861)	0.101	0.689 (0.659–0.719)	0.675 (0.615–0.734)	0.68 (0.644–0.717)	0.870	< 0.001
**Severe sarcopenia**									
EWGSOP2	0.787 (0.742–0.831)	0.761 (0.683–0.838)	0.798 (0.744–0.851)	0.438	0.795 (0.755–0.834)	0.815 (0.744–0.885)	0.774 (0.727–0.822)	0.354	0.709
AWGS2019	0.737 (0.697–0.778)	0.739 (0.679–0.798)	0.743 (0.690–0.796)	0.922	0.858 (0.831–0.885)	0.85 (0.805–0.894)	0.871 (0.842–0.899)	0.436	< 0.001

Notes Data are presented with the 95% confidence interval in parenthesis. *P*_*1*_, *P* value for equal AUC between genders. *P*_*2*_, *P* value for equal AUC between SARC-F and SARC-CalF in total population

Abbreviations AUC, the area under the receiver operating characteristics curve; EWGSOP2, European Working Group on Sarcopenia in Older People; AWGS2019, Asian Working Group for Sarcopenia; IWGS, the International Working Group on Sarcopenia; FNIH, the Foundation for the National Institutes of Health Biomarkers Consortium; SDOC, the Sarcopenia Definition and Outcomes Consortium

#### Diagnostic tests of SARC-F or SARC-CalF with different cut-off values

Table 3 outlines the results of SARC-F diagnostic tests against the diagnostic criteria of sarcopenia and severe sarcopenia, including sensitivity, specificity, PPV, NPV and Youden index with different cut-off values. With an initial cut-off point of ≥ 4 to screen sarcopenia, the SARC-F tool demonstrated low sensitivity below 20%, except for of FNIH and SDOC definitions, where it reached 37.9% and 32.4%, while maintaining high specificity of over 90%. For severe sarcopenia, the SARC-F showed increased sensitivity of 38.2% and 24.4% under the definitions of EWGSOP2 and AWGS2019, compared to confirmed sarcopenia screening.

As shown in Table S3, SARC-CalF had moderate sensitivity of 32.6%, 40.7% and 47.6% when using the initial cut-off point of ≥ 11, against the definitions of EWGSOP2, AWGS2019 and IWGS, respectively.

**Table 3:** The results of SARC-F diagnostic tests with different cut-off values against sarcopenia and severe sarcopenia

Diagnostic criteria	Cut-off	Sensitivity	Specificity	PPV	NPV	Youden index
**Sarcopenia**						
EWGSOP2	≥ 2	36.6 (32.0-41.4)	84.3 (82.3–86.1)	40.7 (35.7–45.8)	81.9 (79.8–83.8)	0.21
	≥ 3	20.6 (16.8–24.7)	91.2 (89.6–92.6)	40.7 (34.0-47.6)	79.6 (77.5–81.5)	0.12
	≥ 4	13.7 (10.6–17.4)	95.1 (93.9–96.2)	45.3 (36.5–54.3)	78.9 (76.9–80.8)	0.09
AWGS2019	≥ 2	37.1 (31.6–42.8)	82.7 (80.8–84.6)	29.4 (24.9–34.2)	87.1 (85.3–88.8)	0.20
	≥ 3	22.5 (17.9–27.7)	90.6 (89.1–92.0)	31.8 (25.6–38.5)	85.8 (84.0-87.4)	0.13
	≥ 4	15.6 (11.7–20.2)	94.8 (93.6–95.8)	36.7 (28.4–45.7)	85.3 (83.5–86.9)	0.10
IWGS	≥ 2	46.2 (39.6–53.0)	83.0 (81.1–84.8)	27.3 (22.9–32.1)	91.8 (90.3–93.2)	0.29
	≥ 3	29.3 (23.5–35.8)	90.9 (89.4–92.3)	30.8 (24.7–37.5)	90.3 (88.8–91.7)	0.20
	≥ 4	19.1 (14.2–24.9)	94.8 (93.6–95.8)	33.6 (25.5–42.5)	89.5 (87.9–90.9)	0.14
FNIH	≥ 2	68.3 (61.8–74.3)	86.1 (84.3–87.7)	40.2 (35.2–45.3)	95.2 (94.0-96.2)	0.54
	≥ 3	50.0 (43.3–56.7)	93.8 (92.5–94.9)	52.3 (45.4–59.2)	93.2 (91.9–94.4)	0.44
	≥ 4	37.9 (31.6–44.7)	97.4 (96.5–98.1)	66.4 (57.5–74.5)	92.0 (90.6–93.2)	0.35
SDOC	≥ 2	65.8 (60.0-71.3)	87.7 (86.0-89.3)	49.1 (44.0-54.2)	93.4 (92.1–94.6)	0.54
	≥ 3	45.1 (39.2–51.1)	94.5 (93.3–95.6)	59.8 (52.9–66.4)	90.5 (89.0-91.9)	0.40
	≥ 4	32.4 (27.0-38.2)	97.7 (96.8–98.4)	71.9 (63.2–79.5)	88.9 (87.3–90.3)	0.30
**Severe sarcopenia**						
EWGSOP2	≥ 2	65.9 (56.8–74.2)	82.7 (80.9–84.5)	21.3 (17.3–25.7)	97.2 (96.2–97.9)	0.49
	≥ 3	45.5 (36.5–54.8)	90.9 (89.4–92.2)	26.2 (20.4–32.6)	95.9 (94.9–96.8)	0.36
	≥ 4	38.2 (29.6–47.4)	95.3 (94.2–96.3)	36.7 (28.4–45.7)	95.6 (94.5–96.5)	0.34
AWGS2019	≥ 2	56.5 (48.7–64.2)	83.1 (81.2–84.8)	24.9 (20.7–29.6)	95.1 (93.8–96.1)	0.40
	≥ 3	34.5 (27.4–42.2)	90.8 (89.3–92.1)	27.1 (21.3–33.6)	93.3 (92.0-94.5)	0.25
	≥ 4	24.4 (18.1–31.6)	94.9 (93.7–95.9)	32.0 (24.1–40.9)	92.7 (91.3–93.8)	0.19

Notes Data are presented with the 95% confidence interval in parenthesis

Abbreviations PPV, Positive predictive value; NPV, Negative predictive value; EWGSOP2, European Working Group on Sarcopenia in Older People; AWGS2019, Asian Working Group for Sarcopenia; IWGS, The International Working Group on Sarcopenia; FNIH, The Foundation for the National Institutes of Health Biomarkers Consortium; SDOC, The Sarcopenia Definition and Outcomes Consortium

**Fig. 3:**
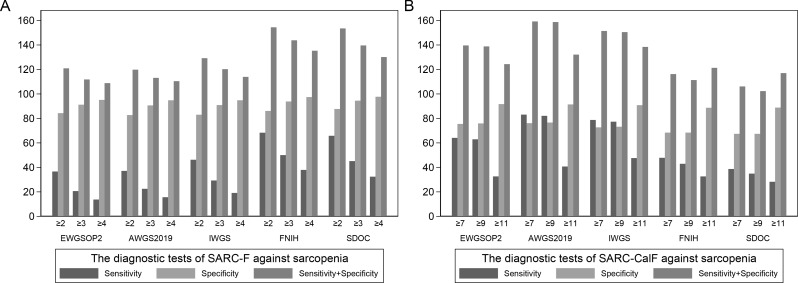
**A** The sensitivity, specificity and sensitivity plus specificity (%) of SARC-F with different cut-off values against the five diagnostic criteria of sarcopenia. **B** The sensitivity, specificity and sensitivity plus specificity (%) of SARC-CalF with different cut-off values against the five diagnostic criteria of sarcopenia (Table S3 for data of all cut-off values)

Figure 3 illustrates the sensitivity and specificity to screen sarcopenia as bars, and the sum of sensitivity and specificity was represented as Youden index. For SARC-F, higher sensitivity and Youden index were observed when using FNIH and SDOC criteria, whereas for SARC-CalF, higher sensitivity and Youden index were found using AWGS2019 and IWGS criteria. Figure S1 provides the additional comparisons of sensitivity and specificity for both tools in screening severe sarcopenia.

To determine the optimal cut-off point, Fig. 3 also displays the diagnostic performance of SARC-F and SARC-CalF with different cut-off values. Using the AWGS2019 sarcopenia criteria, SARC-F achieved a maximum Youden index of 0.20 at a cut-off point of ≥ 2, while SARC-CalF reached a maximum Youden index of 0.60 at a cut-off of ≥ 7. At the cut-off of ≥ 2, the Chinese version SARC-F yielded a positive rate of 20.49% with enhanced moderate sensitivity for both confirmed and severe sarcopenia, as detailed in Table 3. At the cut-off of ≥ 7, the Chinese version SARC-CalF had a positive rate of 33.51%, with enhanced sensitivity of 83.1% and 84.5%, and specificity of 70.7% and 71.6% respectively, against AWGS2019 definition of confirmed and severe sarcopenia, as shown in Table S3.

## Discussion

In this study, we obtained the Chinese translated and cultural adaption version of SARC-F questionnaire, which proved to be time-saving and well-received by Chinese population. Through the clinical validation of 1859 seniors in communities in China, we observed that SARC-F and SARC-CalF had reasonable diagnostic accuracy for screening sarcopenia in both males and females. Our results revealed low sensitivity and high specificity against five different definitions of sarcopenia using cut-off point of the original SARC-F tool. It was also evident that SARC-CalF could significantly enhance the diagnostic accuracy of SARC-F. To determine the optimal cut-off point of Chinese version SARC-F and SARC-CalF, we exploratively set a score ≥ 2 for SARC-F and a score ≥ 7 for SARC-CalF to classify older individuals as at risk of sarcopenia in Chinese population.

Nowadays, the operational definition of sarcopenia has not reached an agreement globally. In our study population, the prevalence of sarcopenia ranged from 12.05 to 22.75%. Against the definition of EWGSOP2, AWGS2019 and IWGS, the prevalence of sarcopenia in males was higher than in females. The prevalence and trend of gender differentiation were similar to previous evidence among Chinese community-dwelling population [20, 21]. To perform a comprehensive exploration, we carried out the analysis against several commonly used sarcopenia definitions, including EWGSOP2, AWGS2019, IWGS, FNIH and SDOC. In the ROC analysis, we observed higher AUCs of SARC-F when using FNIH and SDOC definitions as references. SDOC definition determines sarcopenia by weakness and slowness, which are in accordance with the five questions of SARC-F mainly focus on muscle function. The choice between height-squared adjusted parameters and BMI-adjusted parameters remains a matter of debate, although the latter had a stronger capacity to identify sarcopenia patients who were more obese [22, 23]. The use of BMI-adjusted parameters in FNIH and SDOC definitions and its association to SARC-F tool needs to be further investigated. We selected the AWGS2019 definition as the referenced definition to determine the optimal cut-off points, as it was recommended by Chinese expert consensus for use among Chinese older populations [24] and was most commonly used in Chinese clinical and research settings.

The SARC-F always exhibited lower sensitivity, higher specificity, lower PPV and higher NPV, which were more or less similar to the results in previous validation studies [19] as well as the translated version in other languages [25–28]. That implies more missed diagnoses and fewer misdiagnoses would occur during sarcopenia screening. Despite the limitations in screening accuracy, the SARC-F remains useful taking advantage of its simplicity and the representativeness of muscle-related adverse outcomes. Our Spearman’s correlation coefficient analysis indicated that the Chinese version SARC-F had good consistency with muscle-related outcome indicators, including muscle mass represented by ASMI, muscle strength represented by grip strength, and physical function represented by gait speed. That suggests the five questions in SARC-F could represent the muscle status to some extent. Besides, the Chinese version SARC-F score was significantly associated with worse nutritional or frailty status in the community-dwelling senior population. Previous studies demonstrated the predictive value of the SARC-F questionnaire for outcomes such as quality of life, hospitalization and mortality, both in the older population [29–31] and in populations with diseases [32–34].

In our study, SARC-F consistently had higher sensitivity and NPV in detecting severe sarcopenia than confirmed sarcopenia. That means SARC-F would mostly detect severe cases but not be sensitive enough to the early changes in muscle. In the consensus of AWGS2019, SARC-F was recommended for use in the referral of at-risk populations for definitive diagnosis [7]. Although we found the potential use of SARC-F in severe sarcopenia, the validation of SARC-F for testing severe sarcopenia has been rare up to now. Globally, SARC-F may be a useful tool for briefly ruling out sarcopenia, but the limitation of screening accuracy may restrict its practical application.

Taking into consideration that the initial cut-off point of SARC-F may not suit for the Chinese population due to the ethnic and cultural differences, we attempted to compare the diagnostic capacity of different cut-off points and to determine an optimal one suitable for Chinese population. According to AWGS2019 criteria, changing the cut-off point to ≥ 2 for SARC-F could enhance its sensitivity to 37.1% in screening sarcopenia, and to 56.5% in screening severe sarcopenia. The new cut-off points may enhance the capacity of SARC-F to early detect potential sarcopenia, while they need to be further validated in a larger Chinese population to advance their clinical use.

In consideration of the pros and cons of SARC-F, we implemented this study and included the participants mostly from rural areas, so as to validate its usage in primary medical settings with limited measurement or specialists and with participants of lower education levels. When investigated and administered the SARC-F instrument, the older participants, especially those from rural areas, were hardly familiar with the syndrome sarcopenia, and the questions of SARC-F well explained what sarcopenia would be like. So it is our target to promote its application by participants themselves in the communities and rural areas to increase the awareness on sarcopenia.

To be an ideal screening tool, it is necessary for the instrument to obtain not only simplicity but also sufficient accuracy. SARC-CalF, as an updated version of SARC-F, was also highly associated with muscle-related indicators and AWGS2019 defined sarcopenia in our Spearman’s correlation coefficient analysis. As demonstrated in our study, SARC-CalF presented higher sensitivity and AUC than SARC-F to screen sarcopenia, which was similarly validated by previous studies [35, 36]. Specifically, the SARC-CalF with the cut-off point of ≥ 11 reached a good sensitivity of nearly 50% when screening severe sarcopenia. We also established an optimal cut-off point of ≥ 7 for SARC-CalF to use in Chinese population, which could further increase the sensitivity when screening sarcopenia and severe sarcopenia. In consideration of its simplicity and the improvement in diagnostic accuracy compared to SARC-F, SARC-CalF was more likely to be a suitable screening tool for sarcopenia in clinical use, especially with the cut-off point of ≥ 7.

Although we aimed to provide evidence for the future use of sarcopenia screening tools in Chinese population, there are limitations in current study. We excluded individuals over 85 years old and those with disabilities to maintain the internal validity of the study. However, this exclusion may underestimate the prevalence of sarcopenia and limit the generalizability of our findings. According to international consensus, older age is one of the most important risk factors of sarcopenia [4, 7]. As a meta-analysis demonstrated, the prevalence of sarcopenia increased with age in the Chinese population, reaching 38.1% in the subgroup of 80 years and older [21]. Based on the original validation studies, the SARC-F and SARC-CalF tools are expected to be valid and useful for individuals over 85 years old [5, 9].

All participants in this study were from North China, and the majority were rural residents, which may not be sufficient to extrapolate to the entire Chinese population. Besides, using the left leg for CC measurements may introduce variability in the SARC-CalF score, and the optimal measurement method requires further investigation. Furthermore, this study was based solely on the cross-sectional data, and the prognostic value of SARC-F tools needs further validation. Therefore, future research should include longitudinal studies with a large, diverse sample across a broader age range from different regions of the country, as well as studies specifically focused on disabled populations.

## Conclusions

The Chinese version SARC-F questionnaire showed good reliability and validity for screening sarcopenia. Despite low sensitivity, it is a useful tool to screen severe cases in communities taking advantage of its simplicity. SARC-CalF was more likely to be a suitable screening tool for sarcopenia in clinical use.

## Electronic supplementary material

Below is the link to the electronic supplementary material.

Supplementary Material 1

## Data Availability

No datasets were generated or analysed during the current study.

## Abbreviations

EWGSOP: European Working Group on Sarcopenia in Older People
AWGS: Asian Working Group for Sarcopenia
IWGS: International Working Group on Sarcopenia
FNIH: Foundation for the National Institutes of Health Biomarkers Consortium
SDOC: Sarcopenia Definition and Outcomes Consortium
CC: Calf circumference
MNA-SF: Mini Nutritional Assessment-Short Form
IPAQ: International Physical Activity Questionnaire
M-BIA: Multifrequency bioelectrical impedance analysis
ASMI: Appendicular skeletal muscle mass index
ALM: Appendicular lean mass
BMI: Body mass index
PPV: Positive predictive value
NPV: Negative predictive value
ROC: Receiver operating characteristics
AUC: The area under the ROC curve
ICC: Intraclass correlation coefficient
CI: Confidence interval
IQR: Interquartile ranges
CHD: Coronary heart disease
