# Supplementary material for: The application of Chinese version of SARC-F and SARC-CalF in sarcopenia screening against five definitions: a diagnostic test accuracy study
Source: BMC Geriatr. 2024 Oct 26;24:883. doi: 10.1186/s12877-024-05460-w (PMC11515234; doi:10.1186/s12877-024-05460-w)
Supplement: Supplementary file 1 — Supplementary Material 1 [file 12877_2024_5460_MOESM1_ESM.docx]

**Supplementary Table 1** Chinese translated version of SARC-F questionnaire

**肌肉衰减症筛查5项评分量表中文版**

| 条目 | 问题 | 得分 |
| --- | --- | --- |
| 力量 | 您举起和搬运4.5千克(9斤)^*^的物品有无困难？ | 无困难＝0分  轻度困难＝1分  非常困难或无法完成＝2分 |
| 协助步行 | 您步行穿过整个房间有无困难？ | 无困难＝0分  轻度困难＝1分  非常困难,需要协助或无法完成＝2分 |
| 座椅起身 | 您从椅子或床上起身有无困难？ | 无困难＝0分  轻度困难＝1分  非常困难或需要协助才能完成＝2分 |
| 爬楼梯 | 您爬10个台阶(大约1层)有无困难？ | 无困难＝0分  轻度困难＝1分  非常困难或无法完成＝2分 |
| 跌倒 | 在过去一年里，您跌倒了多少次？ | 无跌倒＝0分  1～3 次＝1分  4次及以上＝2分 |

^*^The weight of 10 pounds was transferred into SI unit (4.5 kilograms), followed by an annotation of 9 jin (a unit of weight used in China, 1 jin=0.5 kilogram).

**Supplementary Table 2** Back translated version of SARC-F questionnaire

**SARC-F: a screening form of sarcopenia**

| Items | Questions | scores |
| --- | --- | --- |
| Strength | Do you have any difficulty of lifting or carrying 4.5kg stuff? | No difficulty=0  Some difficulty=1  A lot of difficulty or cannot do it=2 |
| Walking with assistance | Do you have any difficulty of walking across a room? | No difficulty=0  Some difficulty=1  A lot of difficulty, need assistance, or cannot do it=2 |
| Getting rise from a chair | Do you have any difficulty of getting rise from a chair or bed? | No difficulty=0  Some difficulty=1  A lot of difficulty, need assistance, or cannot do it=2 |
| Stair climbing | Do you have any difficulty of climbing a flight of 10 steps? | No difficulty=0  Some difficulty=1  A lot of difficulty, need assistance, or cannot do it=2 |
| Falls | How many times of falling down in the past year? | No fall=0  1~3times=1  At and above 4 times=2 |

**Supplementary Table 3** The results of SARC-CalF diagnostic tests with different cut-off values against sarcopenia and severe sarcopenia

| **Diagnostic criteria** | **Cut-off** | **Sensitivity** | **Specificity** | **PPV** | **NPV** | **Youden index** |
| --- | --- | --- | --- | --- | --- | --- |
| Sarcopenia |  |  |  |  |  |  |
| EWGSOP2 | ≥7 | 64.1 (59.3-68.6) | 75.5 (73.2-77.7) | 43.5 (39.6-47.5) | 87.7 (85.7-89.5) | 0.4 |
|  | ≥8 | 63.4 (58.6-68.0) | 75.8 (73.5-78.0) | 43.6 (39.6-47.6) | 87.5 (85.6-89.3) | 0.39 |
|  | ≥9 | 62.9 (58.1-67.5) | 75.9 (73.6-78.1) | 43.5 (39.5-47.5) | 87.4 (85.4-89.2) | 0.39 |
|  | ≥10 | 62.6 (57.8-67.3) | 75.9 (73.6-78.1) | 43.4 (39.4-47.4) | 87.3 (85.4-89.1) | 0.39 |
|  | ≥11 | 32.6 (28.2-37.3) | 91.7 (90.2-93.1) | 53.7 (47.4-59.9) | 82.2 (80.2-84.1) | 0.24 |
| AWGS2019 | ≥7 | 83.1 (78.4-87.2) | 76.1 (73.9-78.2) | 40.3 (36.4-44.3) | 95.9 (94.6-96.9) | 0.59 |
|  | ≥8 | 82.5 (77.7-86.6) | 76.5 (74.3-78.6) | 40.5 (36.6-44.5) | 95.7 (94.5-96.8) | 0.59 |
|  | ≥9 | 82.1 (77.3-86.3) | 76.6 (74.4-78.7) | 40.5 (36.6-44.5) | 95.7 (94.4-96.7) | 0.59 |
|  | ≥10 | 81.8 (77.0-86.0) | 76.6 (74.4-78.7) | 40.4 (36.5-44.4) | 95.6 (94.3-96.7) | 0.58 |
|  | ≥11 | 40.7 (35.1-46.5) | 91.4 (89.9-92.7) | 47.9 (41.6-54.2) | 88.8 (87.2-90.3) | 0.32 |
| IWGS | ≥7 | 78.7 (72.7-83.8) | 72.7 (70.5-74.9) | 28.4 (24.9-32.1) | 96.1 (94.9-97.1) | 0.51 |
|  | ≥8 | 77.8 (71.8-83.0) | 73.1 (70.9-75.2) | 28.5 (24.9-32.2) | 96 (94.7-97.0) | 0.51 |
|  | ≥9 | 77.3 (71.3-82.6) | 73.2 (71-75.3) | 28.4 (24.9-32.2) | 95.9 (94.7-96.9) | 0.51 |
|  | ≥10 | 76.9 (70.8-82.2) | 73.2 (71.0-75.3) | 28.3 (24.8-32.1) | 95.8 (94.6-96.9) | 0.5 |
|  | ≥11 | 47.6 (40.9-54.3) | 90.8 (89.3-92.2) | 41.6 (35.5-47.9) | 92.6 (91.2-93.9) | 0.38 |
| FNIH | ≥7 | 47.8 (41.1-54.5) | 68.4 (66.1-70.7) | 17.2 (14.3-20.4) | 90.5 (88.8-92.1) | 0.16 |
|  | ≥8 | 44.2 (37.6-51.0) | 68.4 (66.1-70.7) | 16.1 (13.3-19.2) | 90 (88.1-91.6) | 0.13 |
|  | ≥9 | 42.9 (36.3-49.6) | 68.4 (66.1-70.7) | 15.7 (12.9-18.8) | 89.7 (87.9-91.4) | 0.11 |
|  | ≥10 | 42.4 (35.9-49.2) | 68.4 (66.1-70.7) | 15.5 (12.8-18.7) | 89.7 (87.8-91.3) | 0.11 |
|  | ≥11 | 32.6 (26.5-39.2) | 88.7 (87.1-90.2) | 28.4 (23-34.3) | 90.6 (89-92) | 0.21 |
| SDOC | ≥7 | 38.7 (33-44.7) | 67.4 (65.1-69.7) | 17.7 (14.7-20.9) | 85.9 (83.9-87.8) | 0.06 |
|  | ≥8 | 35.9 (31.3-41.8) | 67.4 (64.1-69.7) | 16.6 (13.7-19.8) | 85.4 (83.3-87.3) | 0.03 |
|  | ≥9 | 34.9 (29.3-40.7) | 67.4 (65.1-69.7) | 16.2 (13.3-19.3) | 85.2 (83.1-87.1) | 0.02 |
|  | ≥10 | 34.5 (29.0-40.4) | 67.4 (65.1-69.7) | 16 (13.2-19.2) | 85.1 (83.0-87.0) | 0.02 |
|  | ≥11 | 28.2 (23-33.8) | 88.8 (87.1-90.3) | 31.1 (25.5-37.2) | 87.3 (85.5-88.9) | 0.17 |
| Severe sarcopenia |  |  |  |  |  |  |
| EWGSOP2 | ≥7 | 69.1 (60.1-77.1) | 69 (66.8-71.2) | 13.6 (11-16.6) | 96.9 (95.8-97.8) | 0.38 |
|  | ≥8 | 66.7 (57.6-74.9) | 69.3 (67.1-71.5) | 13.3 (10.7-16.3) | 96.7 (95.6-97.6) | 0.36 |
|  | ≥9 | 65 (55.9-73.4) | 69.4 (67.1-71.5) | 13.1 (10.5-16) | 96.6 (95.4-97.5) | 0.34 |
|  | ≥10 | 64.2 (55.1-72.7) | 69.4 (67.1-71.5) | 12.9 (10.4-15.9) | 96.5 (95.3-97.4) | 0.34 |
|  | ≥11 | 49.6 (40.5-58.8) | 88.7 (87.1-90.2) | 23.7 (18.7-29.4) | 96.1 (95.1-97) | 0.38 |
| AWGS2019 | ≥7 | 84.5 (78.2-89.6) | 71.6 (69.3-73.7) | 22.8 (19.6-26.3) | 97.9 (96.9-98.6) | 0.56 |
|  | ≥8 | 83.3 (76.8-88.6) | 71.9 (69.7-74.0) | 22.8 (19.5-26.3) | 97.7 (96.8-98.5) | 0.55 |
|  | ≥9 | 82.7 (76.2-88.1) | 72 (69.8-74.2) | 22.7 (19.4-26.2) | 97.7 (96.7-98.4) | 0.55 |
|  | ≥10 | 82.1 (75.5-87.6) | 72 (69.8-74.2) | 22.6 (19.3-26.1) | 97.6 (96.6-98.4) | 0.54 |
|  | ≥11 | 57.7 (49.9-65.3) | 90.5 (89.0-91.9) | 37.7 (31.8-44.0) | 95.6 (94.4-96.5) |  |

Notes: Data are presented with the 95% CI in parenthesis.

Abbreviations: PPV, positive predictive value; NPV, negative predictive value; EWGSOP2, European Working Group on Sarcopenia in Older People; AWGS2019, Asian Working Group for Sarcopenia; IWGS, the International Working Group on Sarcopenia; FNIH, the Foundation for the National Institutes of Health Biomarkers Consortium; SDOC, the Sarcopenia Definition and Outcomes Consortium.

**Supplementary Figure 1**


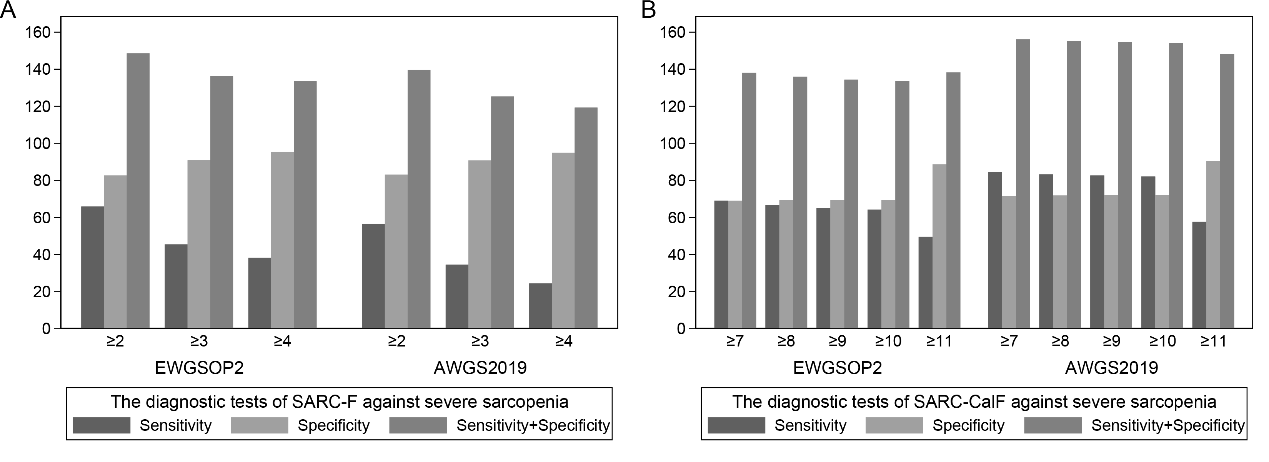


Figure S1 A: The sensitivity, specificity and sensitivity plus specificity (%) of SARC-F with different cut-off values against severe sarcopenia. B: The sensitivity, specificity and sensitivity plus specificity (%) of SARC-CalF with different cut-off values against severe sarcopenia.
